# Elevation-associated shifts in plasma metabolite abundance and lung gene expression in the Xizang plateau frog, *Nanorana parkeri*

**DOI:** 10.1186/s12864-026-12553-w

**Published:** 2026-01-20

**Authors:** Xuejing Zhang, Yonggang Niu, Shengkang Men, Qiang Chen, Xiaolong Tang

**Affiliations:** 1 School of Life Sciences, Lanzhou University, Lanzhou, Gansu 730000 China; 2 School of Life Sciences, Dezhou University, Dezhou, Shandong 253023 China

**Keywords:** *Nanorana parkeri*, High elevation, Adaptations, Metabolome, Transcriptome, Metabolic flux

## Abstract

**Background:**

Compared to other amphibians, the Xizang plateau frog, *Nanorana parkeri*, is the highest elevation-dwelling amphibian species known to date (up to 5,100 m), offering a valuable model for understanding ectotherm adaptation to extreme environments. Here, we compared plasma metabolomes and lung transcriptomes of frogs between higher (4,600 m) and lower (3,400 m) elevations. We also assayed key metabolites (glucose, lactate, NADH, β-hydroxybutyrate) in the plasma and inferred the metabolic flux of central metabolic pathways.

**Results:**

Plasma metabolomics revealed significant elevation-related differences, identifying 222 differential metabolites. High-elevation frogs exhibited 37% higher glucose but 32% and 33% lower lactate and β-hydroxybutyrate, respectively, alongside reduced glycolytic and fatty acid metabolism fluxes. Lung transcriptomic analysis identified 1,618 differentially expressed genes, with broad down-regulation of glycolysis, TCA cycle, oxidative phosphorylation, fatty acid oxidation, and PPAR signaling in high-elevation frogs, indicating metabolic rate depression. Canonical hypoxia sensors (*HIF1A*, *EGLN1-3*) showed no differential expression, but transcription factors (*ATF3*, *JUN*, *ARNT2*) and stress-response pathways (Wnt, MAPK, and G protein-coupled receptor signaling) were up-regulated in high-elevation frogs. Increased expression of fibroblast growth factors and *IGFBP2* in high-elevation individuals may indicate vascular remodeling. At higher elevation, the up-regulation of potassium/calcium channels, TRP channels, and aquaporins (*AQP1*, *AQP4*) may be linked to ion and water homeostasis. Moreover, higher expression of DNA repair-related genes (*RAD18*, *RAD51*), heat shock proteins (*HSPB6*, *HSP40*), and adhesion molecules (*ADAM22*, cadherins) was consistent with enhanced cellular stress tolerance under high-elevation conditions.

**Conclusions:**

These results reveal that *N. parkeri* shows coordinated shifts in metabolite abundance and gene expression associated with higher elevation, providing new insights into molecular mechanisms of ectotherm adaptation to extreme environments.

**Supplementary Information:**

The online version contains supplementary material available at 10.1186/s12864-026-12553-w.

## Background

Ectothermic vertebrates living at high elevations experience multiple environmental challenges, including chronic hypoxia, low temperature, and intense ultraviolet radiation (UVR). These stressors impose strong constraints on aerobic metabolism and homeostatic balance, driving the evolution of physiological and molecular strategies such as metabolic rate depression, antioxidant defense enhancement, and stress protein induction [1, 2]. Amphibians inhabiting the Qinghai-Xizang Plateau serve as natural models for exploring these adaptive mechanisms. Among them, the Xizang plateau frog (*Nanorana parkeri*), distributed across elevations from 2,850 to 5,100 m, provides a good opportunity to examine how ectothermic vertebrates cope with extreme high-elevation environments [3, 4]. The physiological ecology and molecular mechanisms underlying its winter hibernation have been well documented. Morphological evidence indicates that frogs gain weight and accumulate glycogen during hibernation [5]. At the physiological level, metabolic rate depression of hibernating frogs occurs independently of temperature, with suppressed antioxidant defenses but enhanced plasma bactericidal capacity [6, 7]. Molecular evidence has shown down-regulation of energy metabolism genes and up-regulation of immune defense genes, with reversible protein phosphorylation playing a key regulatory role [8, 9]. In addition, high-elevation individuals display higher hemoglobin concentration, greater erythrocyte counts, and improved antioxidant defenses [10]. We recently found that symbiotic gut microbes also play an important role in facilitating the adaptation of *N. parkeri* frogs to extreme environments at high elevations. Two butyrate-producing bacterial genera (*Anaerovorax* and *Pygmaiobacter*) and families such as antimicrobial GH90 and GT103, associated with inflammatory attenuation, were significantly up-regulated in high-elevation individuals [11]. However, the metabolic shifts and gene expression patterns associated with high elevation in *N. parkeri* remain poorly characterized.

Overwhelming evidence suggests that a concomitant reduction in metabolic rate and metabolic demand represents conservative and critical adaptations of ectotherms to environmental stresses such as long-term cold, hypoxia, freezing, and dehydration [1, 12, 13]. The extreme stresses in high-elevation environments are no exception, and native species have evolved physiological and molecular strategies, including cellular and organ-level metabolic remodeling [14, 15]. Such remodeling typically involves: (1) a shift toward anaerobic metabolism coordinated by oxygen and/or energy charge sensors; (2) accumulation of fermentable substrates (e.g., glycogen) to prolong survival; and (3) down-regulation of energy-consuming processes including ionic conductance (ionic pumping) and protein synthesis [2]. High-elevation ectotherms can tolerate metabolic byproducts (e.g., lactate) accumulation and employ enhanced antioxidant defenses to mitigate reactive oxygen species (ROS) generated during hypoxia and reoxygenation [2, 10, 14]. The genes driving these physiological strategies are finely regulated at different levels (e.g. transcriptional and post-transcriptional). For instance, genes linked to energy metabolism generally showed lower mRNA levels in high-elevation species compared to low-elevation counterparts [16]. Hypoxia-inducible factors (HIFs), a well-studied hypoxia response mechanism, consist of a regulated HIFα protein subunit (expressed only during hypoxia) and a constitutively expressed HIF1β protein subunit [17]. HIF transcription factors bind to hypoxia-responsive elements within promoter regions of target genes involved in cell survival, erythropoiesis, angiogenesis, autophagy, and energy metabolism, such as vascular endothelial growth factor (*VEGF*) and glucose transporter (*GLUT*), thereby regulating their expression [18]. Although HIFs and target gene expression (e.g., *VEGF*, transferrins) in the heart was elevated in high-elevation *N. parkeri* [19], it remains unknown whether the lung, as an important respiratory organ, also coordinates gene expression via HIF pathways for high-elevation adaptations.

Omic technologies, such as transcriptomics, proteomics, and metabolomics, have been widely used in environmental stress physiology, enabling global hypothesis-free screening for molecules linked to stress tolerance and phenotypic plasticity [20]. For instance, transcriptomic analyses have identified a series of candidate genes involved in oxygen transport, UVR response, and free radical damage repair in the plateau brown frog *Rana kukunoris* [21], and revealed a suppressed expression of energy metabolism genes along an altitudinal gradient in *Bufo gargarizans* [14]. Compared to transcriptomics or proteomics, metabolomics provides a more functionally relevant understanding, as metabolite abundance often directly reflects phenotype. For example, in lizards, *Phrynocephalus vlangalii*, comparative metabolomics analysis revealed that high-elevation individuals mainly mobilize carbohydrates for energy supply rather than lipids, and that their membrane lipids undergo adaptive remodeling [15]. Thus, probing high-elevation adaptation from metabolomics and transcriptomics perspectives could help to identify candidate genes and small molecules underpinning stress adaptation and to predict regulatory networks from a holistic perspective.

We hypothesized that energy metabolism in *N. parkeri* is remodeled at high elevation to match energy consumption and energy production, reflecting suppressed metabolic rates in colder environments. Moreover, the transcriptomic profile of the lung, an important respiratory organ directly exposed to hypoxia, is expected to undergo significant changes, such as the up-regulation of genes associated with hypoxia-response signaling pathways and the down-regulation of genes involved in metabolic process and ion channel for energy conservation under reduced oxygen availability. To test these hypotheses, we compared plasma metabolomic and lung transcriptomic profiles of *N. parkeri* between higher (4,600 m) and lower (3,400 m) elevations. Plasma metabolomics captures whole-organism metabolic status, whereas lung transcriptomics reflects tissue-specific transcriptional adjustments. Their integration of these data provides complementary insight into systemic and tissue-specific responses to high-elevation stress. Moreover, plasma small molecules (glucose, lactate, β-hydroxybutyrate (β-HB), and nicotinamide adenine dinucleotide (NADH)) were tested, a metabolic network of the central metabolic pathway (CMP) was constructed, and metabolic fluxes were computationally inferred.

## Methods

### Sample collection

In July 2021, adult male *N. parkeri* frogs (*n* = 28) were captured from two altitudinal zones in Xizang, China: lower elevation (3,400 m; Lulang; 29.69°N, 94.73°E) and higher elevation (4,600 m; Mila Mountain; 29.75°N, 92.31°E). Both populations belong to the *N. parkeri* lineage East [22]. Although both sites are at relatively high elevations, Mila Mountain (4,600 m) has a lower partial oxygen pressure (~ 11.9 kPa) and a lower mean water temperature (12.09 °C) than Lulang (3,400 m; ~14.0 kPa and 20.07 °C, respectively) [10]. Moreover, the mean dissolved oxygen concentration of ponds was 56% lower at high elevation than that at low elevation [23]. Thus, the higher elevation imposes substantially greater physiological challenges to frogs. Because *N. parkeri* is primarily nocturnal, sampling was performed during the daytime (12:00–15:00 h, at least 6 h post-feeding) from resting microhabitats to minimize diet- and activity-related physiological variation. Morphological parameters, including body mass (BM) and snout-vent length (SVL), were measured (Table 1). Frogs were immediately euthanized via spinal cord destruction, and both blood and lung tissues were collected within 3 min to minimize stress-induced metabolic changes. Approximately 100 µL of blood was withdrawn from the common carotid artery using heparinized glass capillary tubes and placed in a heparinized centrifuge tube. All blood samples were immediately centrifuged at 3,000 g for 10 min within 1 min of collection, and the plasma was instantly flash-frozen in liquid nitrogen to prevent NADH oxidation. Moreover, the lung was excised and snap-frozen in liquid nitrogen. All plasma and lung samples were stored at -80 °C until subsequent analyses.

**Table 1:** Morphometric parameters of *N. parkeri* collected from higher and lower elevations

	Lower elevation	Higher elevation
Mean body mass (g)	5.02 ± 0.19	4.75 ± 0.18
Mean snout-vent length (cm)	4.13 ± 0.07	4.08 ± 0.05

The data are expressed as the means ± SEM (*n* = 14)

### Plasma metabolomics analysis

#### Metabolite extraction

After the frozen samples (*n* = 6 per group) were thawed, 50 µL of plasma was pipetted into 300 µL of extraction solution (acetonitrile: methanol = 1:4, V/V, containing 2-chlorophenylalanine as the internal standard), vortexed for 3 min, and centrifuged at 13,800 g for 10 min at 4 °C. Aliquots (200 µL) of supernatants were collected, stored at -20 °C for 30 min, and re-centrifuged (13,800 g, 3 min, 4 °C). Finally, aliquots (180 µL) of supernatants were used for LC-MS analysis. In addition, a 10 µL aliquot of each sample was drawn and mixed to generate a quality control sample.

### LC-MS analysis

Metabolomics analysis was performed using liquid chromatography-mass spectrometry (LC-MS) under both positive and negative ion conditions, consisting of an LC-20 A HPLC (Shimadzu, Japan) coupled to a triple quadrupole-linear ion trap mass spectrometer (TripleTOF 6600+, SCIEX, USA). The analytical conditions were as follows: UPLC column, Waters Acquity UPLC HSS T3 column (1.8 μm, 2.1 mm × 100 mm; Waters, USA); column temperature, 40 °C; flow rate, 0.4 mL min^− 1^; injection volume, 2 µL; solvent system, A: water (0.1% formic acid), B: acetonitrile (0.1% formic acid); gradient program, 5% B at 0 min, 90% B at 11.0 min, 90% B at 12.0 min, 5% B at 12.1 min, 5% B at 14.0 min. The MS conditions were set as follows: ion source gas 1, 50 psi; ion source gas 2, 60 psi; curtain gas, 35 psi; capillary temperature, 550 °C; declustering potential, 60 V (positive mode) and − 60 V (negative mode); collision energy, 30 V (positive mode) and − 30 V (negative mode). Peak extraction, alignment, and retention time correction were conducted using the XCMS program. The peak area was corrected using the support vector regression (SVR) method, and peaks with > 50% missing values were filtered within each group. Corrected peaks were characterized by searching the self-built database (Wuhan Metware Biotechnology Co., Ltd., China), integrated public database, and metDNA. Bioinformatic analysis was conducted using the Metware Cloud platform (https://cloud.metware.cn/#/home). Significantly differential metabolites (SDMs) were identified according to thresholds with *P* < 0.05 from a two-tailed *t*-test and the variable importance for the projection (VIP > 1) from orthogonal partial least squares-discriminant analysis (OPLS-DA).

### Plasma metabolites and metabolic flux analysis

Plasma glucose, lactate, NADH, and β-HB (*n* = 8 per group) were assayed using commercial kits (Nanjing Jiancheng Ltd. Co., Nanjing, China; Beijing Solarbio Science & Technology Co., Ltd., China). Biochemical analysis was performed according to the manufacturer’s instructions (the corresponding product numbers: A154-1-1, A019-2-1, A114-1-1, BC5080). All metabolite levels were expressed as micromoles per milliliter of plasma (µmol mL^−1^ plasma). The metabolic network of CMP was constructed, and metabolic flux analysis was performed as previously reported [14]. Specifically, the CMP network (Supplementary Fig. S1) encompassed ten interconnected reactions that included glycolysis, the pentose phosphate pathway, and the tricarboxylic acid (TCA) cycle. Six key intermediates were assumed to be in pseudo-steady states according to metabolic flux calculation principles [24], including glucose-6-phosphate, fructose-6-phosphate, glyceraldehyde-3-phosphate, phosphoenolpyruvate, pyruvate, and acetyl-CoA. This assumption implies that their accumulation was negligible, leading to zero net change in their respective mass balance equations (Supplementary Table S1). Absolute concentrations of glucose, lactate, β-HB, and NADH were incorporated into the stoichiometric matrix to estimate the metabolic flux for each pathway, which were normalized to the rate of glucose input (r1 set to 100) to enable cross-pathway comparison. A directed, weighted metabolic network diagram was generated to visualize inferred flux distributions, where line thickness represents relative flux magnitude and arrow direction indicates the flow direction of pathways.

### Lung transcriptomics analysis

Total RNA was extracted from lung tissues (*n* = 4 per group) using Trizol reagent (Invitrogen, USA) and tested for its integrity using a Bioanalyzer 2100 system (Agilent Technologies, CA, USA). RNA integrity (RIN value) was assayed using Agilent 5400 system. The mRNA was enriched using poly-T oligo-attached magnetic beads and then fragmented. The first strand of cDNA was synthesized using random oligonucleotides as primers, followed by degradation using RNaseH, and the second strand of cDNA was synthesized. The purified double-stranded cDNA was end-repaired, A-tailed, and ligated into sequencing junctions. The cDNA was screened with AMPure XP beads for cDNAs of 370–420 bp and amplified by PCR. The PCR products were purified to obtain the cDNA library. The Illumina NovaSeq X Plus was used for paired-end 150-bp sequencing. Data quality control, mapping to the genome, and read counting were conducted as previously reported [8]. Differentially expressed genes (DEGs) were identified using the criteria of |Log2 fold change| >1 and a false discovery rate (FDR) < 0.05. Gene ontology (GO) and Kyoto Encyclopedia of Genes and Genomes (KEGG) analyses of DEGs were conducted, and a *P*-value of 0.05 was set as the threshold for significant enrichment. Additionally, gene set enrichment analysis (GSEA v4.3.2, MSigDB) was performed with default parameters to identify predefined gene sets exhibiting significant enrichment between the two elevation groups. Significant enrichment was defined as |normalized enrichment score (NES)| > 1 and nominal *P* < 0.01.

### Statistical analysis

The data from biochemical analyses were examined for normality of distribution and homogeneity of variance and then were analyzed using a Student’s *t*-test. Data are presented as mean ± SEM, and a statistical significance was accepted at *P* < 0.05.

## Results

### Plasma metabolome

**Fig. 1:**
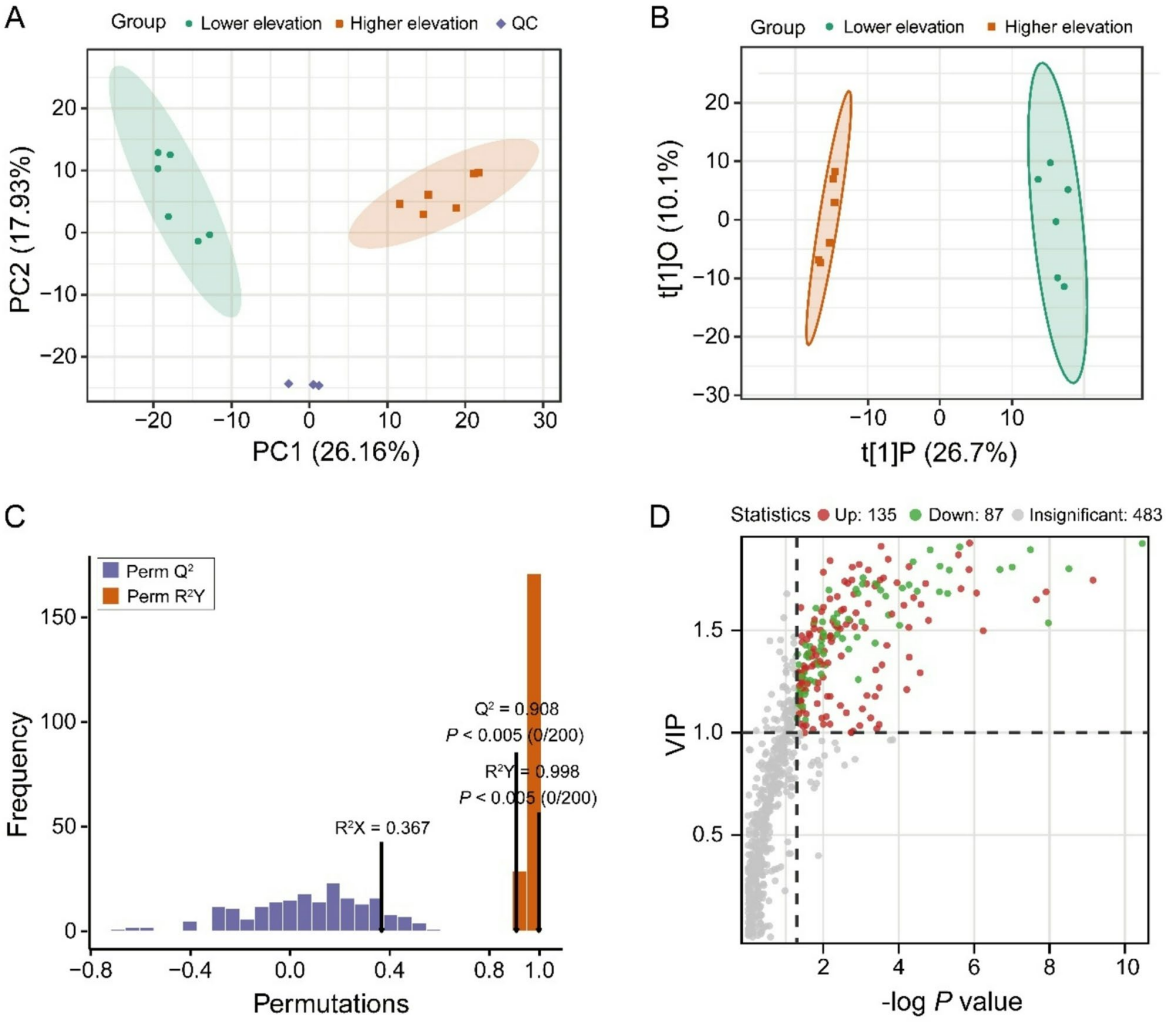
Overview of plasma metabolomic differentiation between high- and low-elevation populations of *N. parkeri. ***A** PCA score plot showing overall clustering of plasma metabolite profiles. Three groups are distinguished: higher elevation (red boxes), lower elevation (green circles), and quality control (QC) samples (purple diamond). **B** OPLS-DA scores plot illustrating separation between high- and low-elevation groups. **C** Permutation tests assessing model robustness and overfitting for the OPLS-DA model. **D** Volcano plot showing changes in plasma metabolites. Red circles represent higher abundance of metabolites, whereas green circles represent lower abundance of metabolites in high-elevation frogs compared with low-elevation individuals

**Fig. 2:**
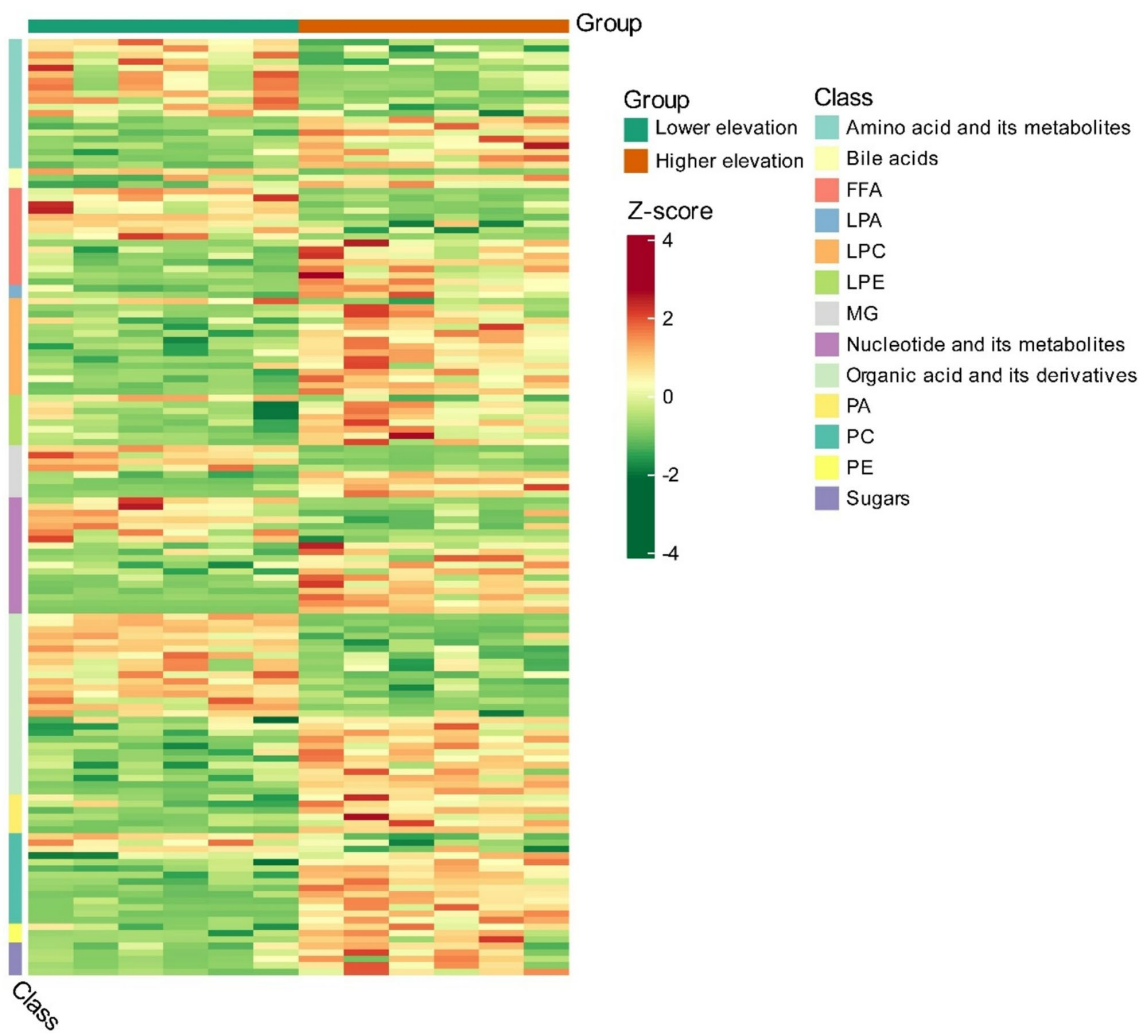
Heatmap visualization of significant differences in plasma metabolite abundance across elevations. Heatmap showing changes in the relative abundance of SDMs and their major classes. Red color indicates metabolites with higher relative abundance, and green indicates low abundance

A principal component analysis (PCA) showed that the two groups (higher elevation vs. lower elevation) were significantly differentiated for the first component (26.16% for PC1) (Fig. 1A). The OPLS-DA model enables the reduction of within-group error, elimination of random error, and identification of representative differential metabolites, which showed a clear distinction between higher and lower elevation groups (Fig. 1B). A permutation test showed that the OPLS-DA parameters R^2^Y and Q^2^ were 0.998 and 0.908, respectively (Fig. 1C), which represented a robust, reliable, and overfitting-free model. A total of 705 metabolites were identified, of which 135 metabolites were up-regulated in higher elevation individuals, whereas 87 metabolites were down-regulated (Fig. 1D; Supplementary Table S2). SDMs were classified into the following classes: amino acids, bile acids, free fatty acids (FFA), lysophosphatidic acid (LPA), lysophosphatidylcholine (LPC), lysophosphatidylethanolamine (LPE), monoglyceride (MG), nucleotides, organic acid, phosphatidic acid (PA), phosphatidylcholine (PC), phosphatidylethanolamine (PE), and sugars (Fig. 2).

### Plasma metabolites and metabolic fluxes

**Fig. 3:**
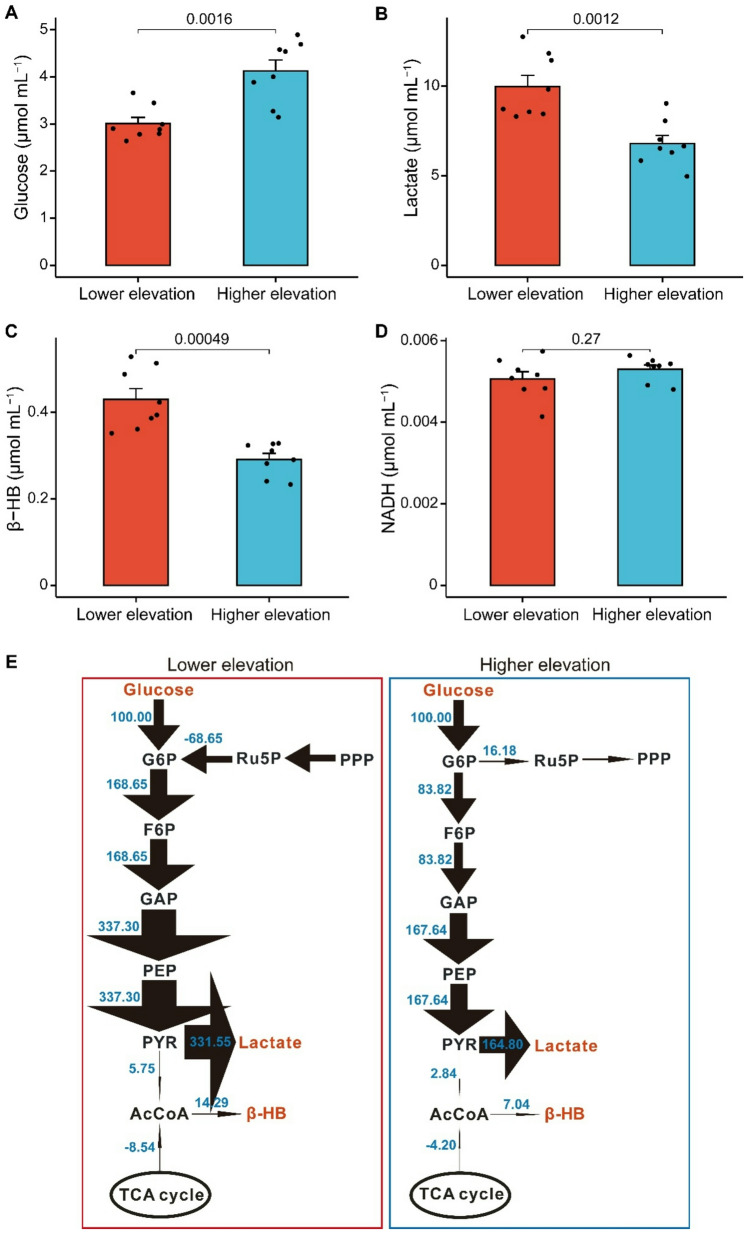
Quantitative analysis of key plasma metabolites and CMP network in *N. parkeri. *Plasma metabolites of *N. parkeri* from higher and lower elevations, including (**A**) glucose, **B** lactate, **C** β-HB, and (**D**) NADH. Data are expressed as means ± SEM (n = 8). **E** A weighted directed network showing metabolic flux of the CMP in plasma of low- and high-elevation frogs. In the network, the line thickness denotes relative flux intensity, and arrows indicate the direction of metabolic reactions. Red-labeled metabolites (glucose, lactate, β-HB, NADH) were quantified experimentally and integrated into the stoichiometric matrix to estimate fluxes. The blue values (normalized to r1: glucose→glucose-6-phosphate, r1 = 100) surrounding the arrows represent relative flux magnitude flowing through the pathway

Glucose content was 37% higher (*t* = -4.182, df = 14, *P* = 0.0016), whereas lactate and β-HB content were 32% (*t* = 4.138, df = 14, *P* = 0.0012) and 33% (*t* = 4.76, df = 14, *P* < 0.001) lower, respectively, in higher elevation frogs than in lower elevation individuals (Fig. 3A–D). Metabolic flux of intermediate metabolites in the glycolytic pathway was lower, and the flux of lactate and β-HB was also reduced in higher elevation frogs compared with those in lower elevation individuals (Fig. 3E).

### Lung transcriptome

#### Transcriptomic data overview and identification of DEGs

**Fig. 4:**
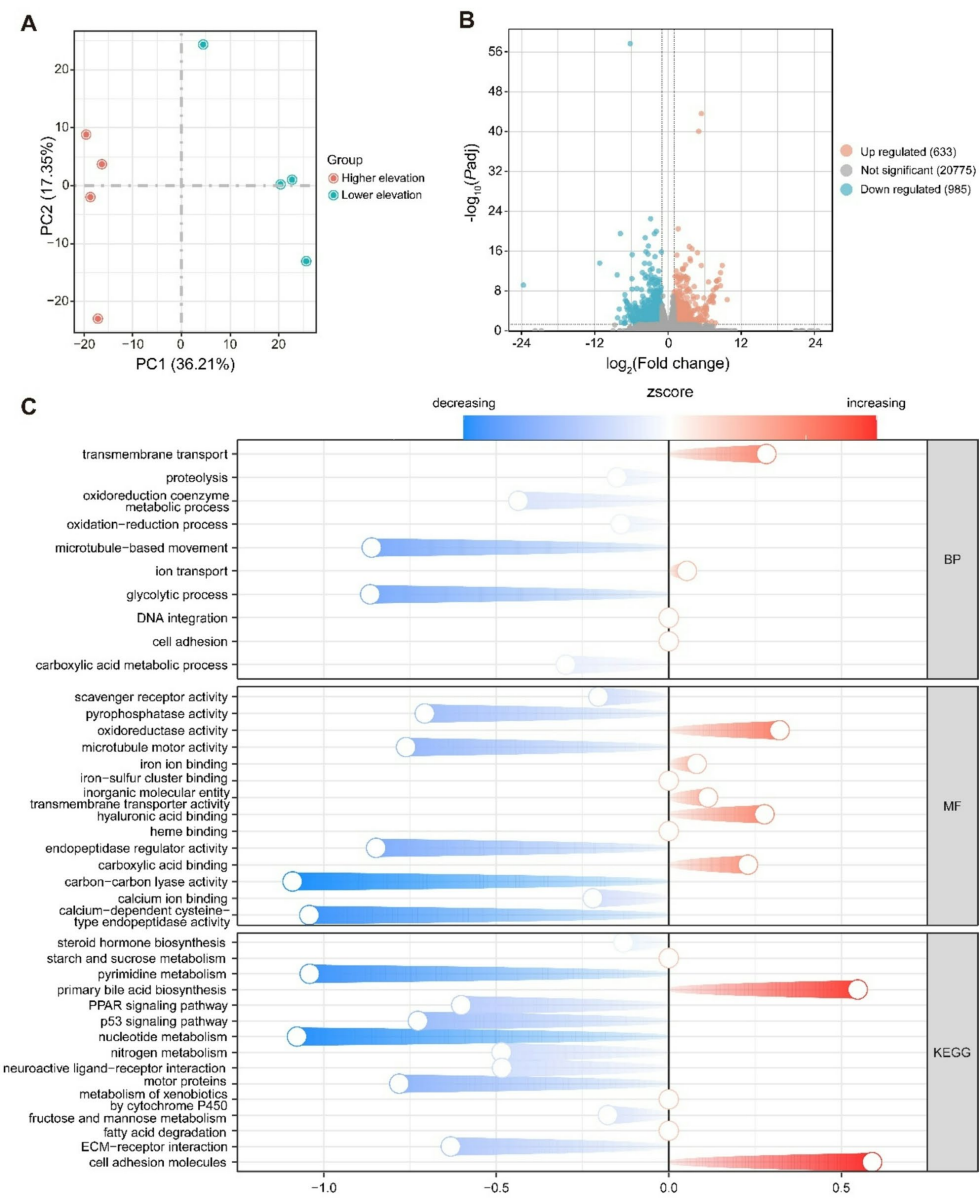
Transcriptomic variation and functional enrichment analysis of DEGs in *N. parkeri. ***A** PCA score plot of lung transcriptome profiles from frogs inhabiting higher (red) and lower (blue) elevations. **B** Volcano plot illustrating DEGs between elevations: red circles represent up-regulated genes, blue circles represent down-regulated genes, and gray circles denote non-differentially expressed genes. **C** Comet plot summarizing significantly enriched GO terms and KEGG pathways of DEGs. Blue color represents down-regulated terms and pathways, and red color indicates up-regulated terms and pathways in high-elevation frogs compared to low-elevation individuals

A total of 50.56 GB of clean data was obtained from eight lung samples, each with > 6.05 GB of clean data. Clean reads matched the reference genome sequence by more than 76.2% (Table 2). Principal component analysis (PCA) showed an obvious distinction between higher and lower elevation samples (Fig. 4A). In total, mRNA expression of 22,393 genes was obtained, of which 985 genes were down-regulated, and 633 genes were up-regulated at higher elevation (Fig. 4B; Supplementary Table S3).

**Table 2:** Summary of RNA-seq quality data of the lung from higher and lower elevations

Sample ID	Clean reads	Clean bases (G)	GC (%)	Q20 (%)	Q30 (%)	Map rate (%)
High_lung1	43,756,546	6.56	44.81	98.31	95.42	86.18
High_lung2	41,194,652	6.18	44.96	98.31	95.38	86.08
High_lung3	40,461,522	6.07	45.20	98.29	95.31	85.58
High_lung4	44,068,910	6.61	45.16	98.33	95.4	85.77
Low_lung1	41,192,294	6.18	45.12	98.34	95.34	76.20
Low_lung2	41,805,870	6.27	45.34	98.38	95.51	84.27
Low_lung3	40,345,332	6.05	44.69	98.30	95.36	84.38
Low_lung4	44,296,346	6.64	44.46	98.40	95.6	77.57

### Functional enrichment analysis of DEGs

**Fig. 5:**
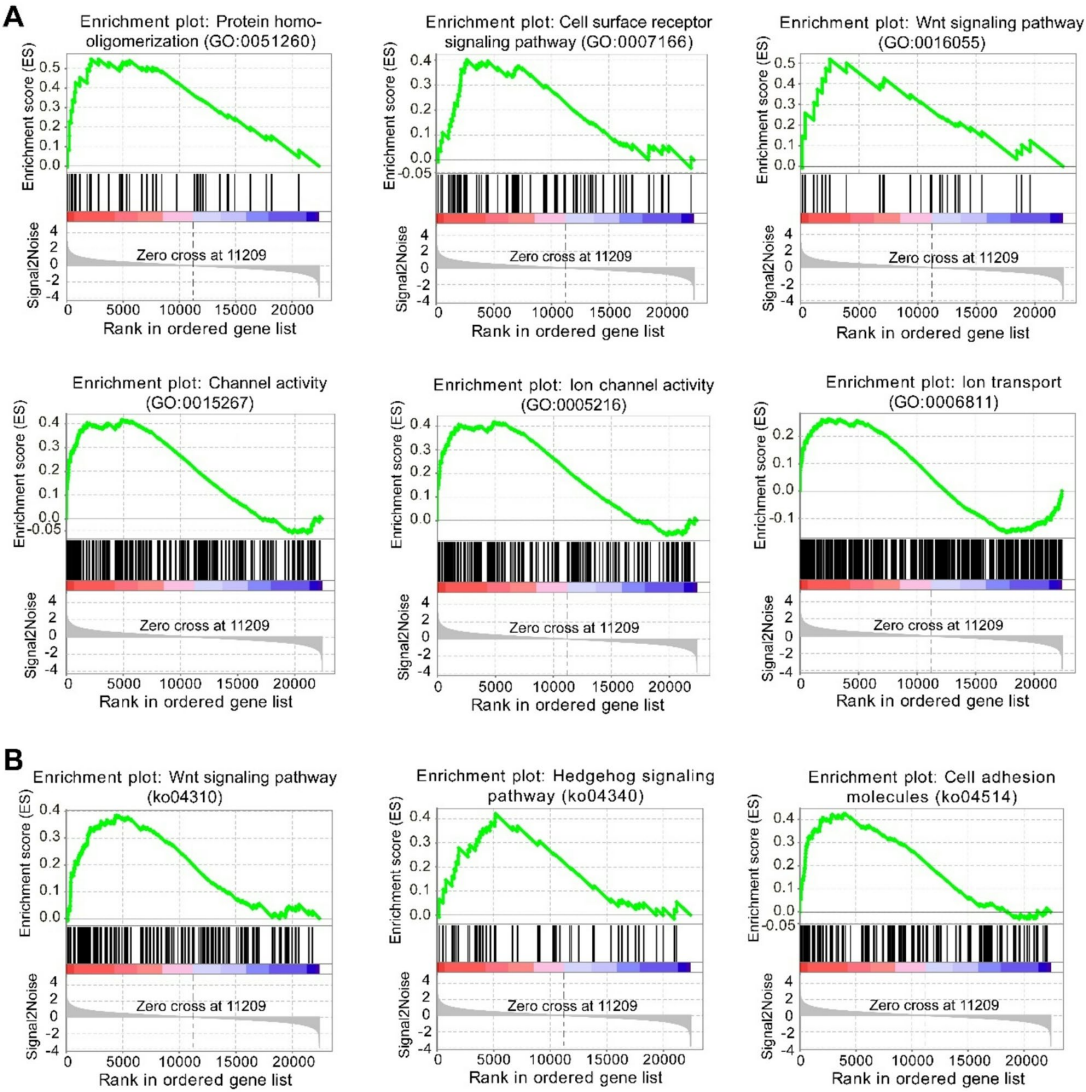
Gene set enrichment analysis (GSEA) of lung transcriptomes in *N. parkeri. ***A** Significantly enriched gene ontology (GO) terms and (**B**) significantly enriched KEGG pathways of GSEA in higher-elevation frogs

GO enrichment analysis showed that DEGs were significantly enriched in microtubule-based movement, oxidation-reduction process, ion transport, carboxylic acid metabolic process, proteolysis, glycolytic process, cell adhesion, oxidoreduction coenzyme metabolic process, transmembrane transport, and DNA integration in the biological process category. Among them, transmembrane transport and ion transport processes were up-regulated, whereas other biological processes were down-regulated except for cell adhesion and DNA integration (Fig. 4C). In the molecular function category, oxidoreductase activity, iron ion binding, inorganic molecular entity transmembrane transporter activity, hyaluronic acid binding, carboxylic acid binding showed a significant up-regulation. Other molecular function showed a significant down-regulation, including endopeptidase regulator activity, scavenger receptor activity, microtubule motor activity, carbon-carbon lyase activity, calcium-dependent cysteine-type endopeptidase activity, pyrophosphatase activity, and calcium ion binding (Fig. 4C). Most of the significantly enriched KEGG pathways were down-regulated, including many metabolic pathways, peroxisome proliferator-activated receptor (PPAR) signaling pathway, and p53 signaling pathway, whereas primary bile acid biosynthesis and cell adhesion molecules were significantly up-regulated (Fig. 4C). GSEA revealed significantly enriched GO terms at higher elevation, including protein homo-oligomerization, cell surface receptor signaling pathway, Wnt signaling pathway, channel activity, ion channel activity, and ion transport (Fig. 5A). In KEGG pathways, GSEA revealed that significant activation of Wnt signaling pathway, hedgehog signaling pathway, and cell adhesion molecules in higher-elevation frogs (Fig. 5B).

### Gene expression correlates of high-elevation adaptation

**Fig. 6:**
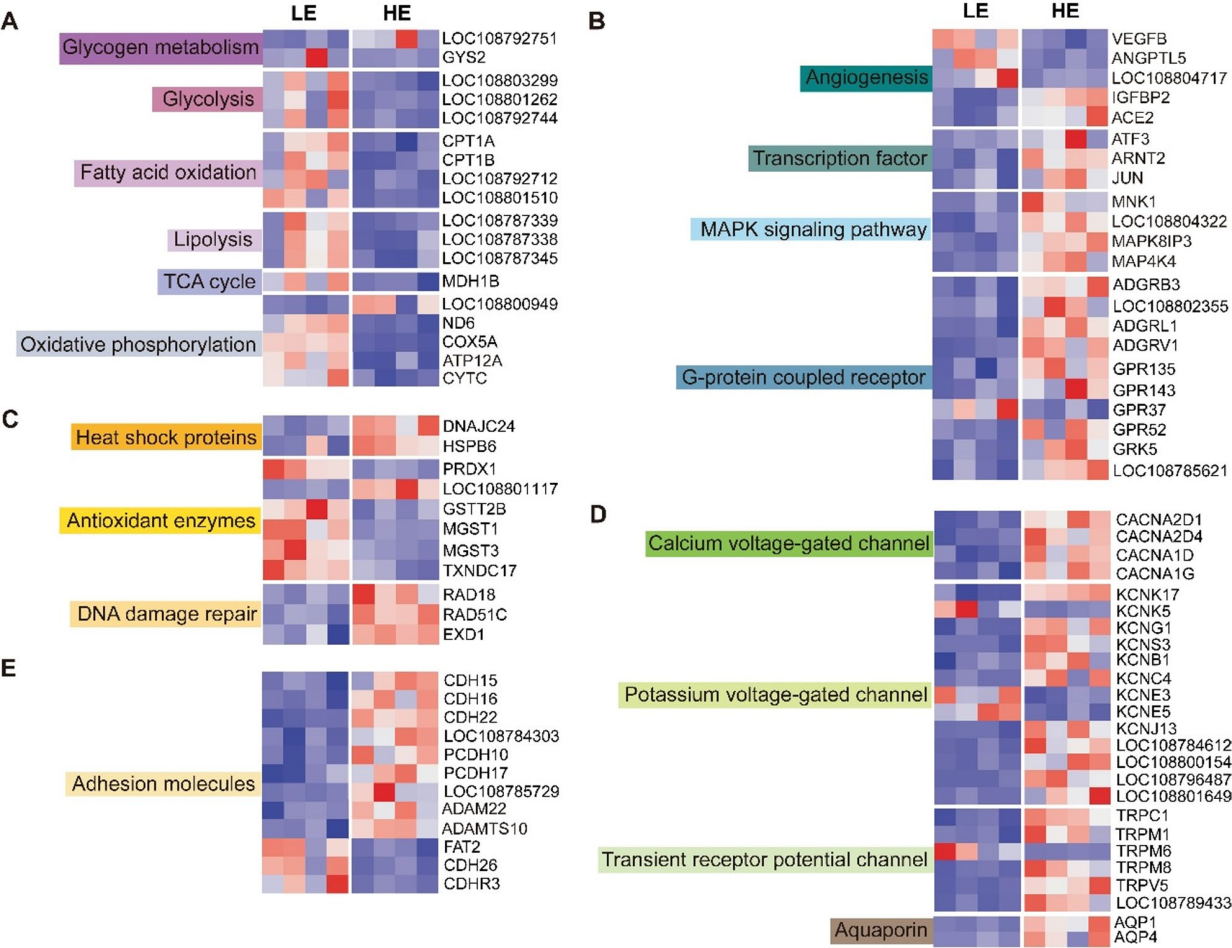
Heatmap visualization of DEGs in lung transcriptomes between high- and low-elevation populations of *N. parkeri. *Heatmaps showing expression changes in genes involved in (**A**) energy metabolism, **B** hypoxia signaling and angiogenesis, **C** stress response and repair, **D** channel proteins, and (**E**) adhesion molecules. LE: lower elevation; HE: higher elevation

Most of the DEGs involved in energy metabolic processes, such as glycolysis, the TCA cycle, fatty acid metabolism, and oxidative phosphorylation, were down-regulated at higher elevation (Fig. 6A). Genes such as *HIF1A*, *EGLN1*, *EGLN2*, and *EGLN3*, encoding hypoxia-inducible factors closely related to hypoxia adaptation, showed no significant differences between the two elevations. Several genes involved in angiogenesis, including *VEGFB* (vascular endothelial growth factor B), *ANGPTL5*, and *LOC108804717* (encoding angiopoietin), were significantly down-regulated at higher elevation. However, genes involved in modulating vascular and cellular signaling in response to hypoxic conditions were significantly up-regulated at higher elevation, including insulin like growth factor binding protein (*IGFBP2*), angiotensin I converting enzyme 2 (*ACE2*), activating transcription factor 3 (*ATF3*), aryl hydrocarbon receptor nuclear translocator 2 (*ARNT2*), AP-1 transcription factor (*JUN*), MAP kinase-interacting serine/threonine-protein kinase (*MNK1*, *LOC108804322*), mitogen-activated protein kinase 8 interacting protein 3 (*MAPK8IP3*), mitogen-activated protein kinase kinase kinase kinase 4 (*MAP4K4*), and G protein-coupled receptor (GPCRs; *ADGRB3*, *LOC108802355*, *ADGRL1*, *ADGRV1*, *GPR135*, *GPR143*, *GPR52*, *GRK5*, *LOC108785621*) (Fig. 6B).

Heat shock proteins, antioxidant enzymes, and genes related to DNA damage repair also showed differential expression between higher and lower elevations (Fig. 6C). Moreover, DEGs encoding calcium and potassium voltage-gated channels, transient receptor potential (TRP) channels, and aquaporin (*AQP1*, *AQP4*) were all greatly up-regulated at higher elevation (Fig. 6D). Genes associated with cell adhesion, including ADAM metallopeptidases (*ADAM22*, *ADAMTS10*), were also up-regulated at higher elevation (Fig. 6E).

## Discussion

Living at high elevation presents significant challenges for amphibians and necessitates physiological and biochemical adaptations for stress tolerance. In this study, comparative analyses of plasma metabolome profiles and lung transcriptome of *N. parkeri* revealed coordinated molecular differences between populations from 3,400 m and 4,600 m. Moreover, DEGs were involved in energy metabolism, hypoxia sensing, stress responses, membrane transport, ion regulation, and extracellular interactions.

The concurrent down-regulation of genes involved in glycolysis, the TCA cycle, and fatty acid oxidation, alongside decreased plasma lactate and β-HB levels, suggests a generalized metabolic rate depression, aligning with prior observations in other high-elevation ectotherms [2, 14, 25, 26]. Our recent study also demonstrated that high-elevation frogs exhibit metabolic rate depression, which was mediated at multiple levels, including resting metabolic rate, liver metabolite content, and enzyme activity [23]. Notably, the mean ambient temperature differed by ~ 8 °C between two sampling sites, a variable that likely influences both metabolic rate and gene expression in ectothermic vertebrates. Thus, the observed down-regulation of energy metabolism pathways could reflect cold-induced metabolic suppression rather than hypoxia-specific responses. Additional factors may also contribute to the detected differences, such as nutritional state, circadian timing, or reproductive condition. PPAR, a lipid-activated transcription factor whose target genes are involved in lipid metabolism, adipogenesis, inflammation, reproduction, cell growth, and differentiation [27], was down-regulated at higher elevation. This down-regulation indicates reduced reliance on oxygen-dependent fatty-acid oxidation and contributes to limiting ROS production. Elevated phospholipids (LPC, LPE, LPA, PC, PE) likely reflect altered membrane composition rather than oxidative energy supply. Enhancing PC and PE levels is particularly beneficial for maintaining membrane structural integrity and fluidity under low-temperature stress, consistent with the principle of homeoviscous adjustment in ectotherms [28]. Moreover, increased bile acid biosynthesis at higher elevation may aid in maintaining energy homeostasis through nuclear receptor and G protein-coupled receptor (GPCR) signaling [29].

The enrichment of oxidation-reduction process likely represents compensatory mechanisms that stabilize redox balance and protect pulmonary tissues from oxidative stress during hypoxia [30]. The significant enrichment of DEGs in microtubule-based movement may indicate cytoskeletal remodeling, as microtubules participate in regulating ciliary beating, mucociliary clearance, and endothelial barrier integrity in the lung [31, 32]. Wnt signaling has been shown to stabilize endothelial function and facilitate angiogenesis under low oxygen tension [33], and Hedgehog signaling promotes alveolar repair and endothelial regeneration [34]. Consequently, the up-regulation of Wnt and Hedgehog signaling pathways at higher elevation may contribute to lung tissue maintenance and vascular remodeling under chronic hypoxia.

As HIF signaling is regulated post-translationally via oxygen-dependent degradation, mRNA levels alone cannot indicate HIF pathway activity [35]. Moreover, HIF1α protein is constitutively transcribed but degraded under normoxia by EGLN (PHD)-mediated hydroxylation. Therefore, mRNA levels of *HIF1A* or *EGLNs* are not expected to change with altitude. Verification of HIF1α protein accumulation, nuclear localization, or target-gene activation will be required to confirm its role in hypoxia response in the lung of *N. parkeri*. *ARNT2* has been identified as a candidate gene for high-elevation adaptation [36]. *JUN* is involved in apoptosis, cell proliferation, inflammation, and angiogenesis, which can be activated by growth factors, pro-inflammatory cytokines, UVR, and hypoxia [37]. *ATF3* is a stress-responsive gene contributing to inhibiting apoptosis, protecting against lung injury, and regeneration [38, 39]. These genes are broadly involved in cellular stress regulation, apoptosis, and tissue maintenance, and thus their up-regulation may point to complementary stress-responsive transcriptional mechanisms.

Although LPA is not a canonical angiogenic factor, its levels were significantly increased at higher elevation. Such change may be beneficial for angiogenesis under hypoxic stress, as these simple lipids participate in cell proliferation and also exert angiogenic effects [40]. Different GPCRs family members have been implicated in LPAs-induced angiogenesis, hypoxia-mediated signaling, and cross talk between *HIF-1* and GPCR-mediated pathways in hypoxia responses [41]. Moreover, *IGFBP2* induces epidermal growth factor receptor signaling and contributes to vascular regeneration [42]. Accordingly, elevated levels of LPAs, along with increased expression of GPCRs, *IGFBP2*, and fibroblast growth factors (*FGF13*, *FGFBP3*, *FGFR4*), may contribute to vascular remodeling in high-elevation frogs. The absence of *VEGF* transcript up-regulation does not preclude VEGF pathway activation via post-translational or receptor-level regulation. Increased *ACE2* expression may help protect against hypoxia-induced lung injury, given its role in preventing pulmonary congestion [43].

Up-regulated MAPK pathway components (*MAPK8IP3*, *MAP4K4*) suggest activation of JNK signaling at higher elevation, which modulates stress responses, angiogenesis, cytoskeletal dynamics, and ion transport [44, 45]. JNK activation under environmental stresses can also stimulate the production of LPAs [46], aligning with the elevated LPA concentrations observed in the plasma at higher elevation. The increased expression of heat shock proteins (Hsp20, Hsp40) may indicate heightened protein quality control under stressful conditions [47]. Moreover, homo-oligomerization is crucial for protein stability, folding, and function [48]. Therefore, activation of protein homo-oligomerization at higher elevation may reflect structural and functional adaptations that enhance protein stability and regulatory flexibility under chronic hypoxia and low-temperature stress. Hsp40 recognizes misfolded proteins in the endoplasmic reticulum lumen and then translocates them to the membrane for ubiquitin-dependent degradation [49]. Coincident with *HSP40* up-regulation, the expression of genes involved in ubiquitination, such as E3 ubiquitin-protein ligase (*TRIM25*, *RNF34*, *TRIM39*, *MUL1*, *NHLRC1*, *RAD18*, *NHLRC1*), was significantly up-regulated. These results allude to the role of posttranslational modifications in regulating the stress response of *N. parkeri*. Interestingly, Rad18 is a DNA damage repair factor that acts within the DNA repair pathway to eliminate UV-induced DNA damage, distinct from classical nucleotide excision repair [50]. Other DNA repair-related genes, such as *RAD51* analogs and *EXD1* (exonuclease 3’–5’ domain containing 1), which mediate repair of DNA double-strand breaks by homologous recombination, were also found to be up-regulated. These results may suggest activation of genome-maintenance processes at higher elevation [51, 52].

Potassium and calcium channels play vital roles in regulating vascular tone and smooth muscle cell proliferation [53, 54]. Thus, their up-regulation at higher elevation potentially contributes to improving blood flow via pulmonary vascular smooth muscle contraction and enhancing oxygen delivery despite theoretical risks of pulmonary hypertension. Intriguingly, TRP channels, including cold-sensing TRPM8, were up-regulated, suggesting transcriptional responses to low temperature at higher elevation. Enhanced ion transport processes at higher elevation may play an important role in maintaining osmotic and ionic balance. The lung expresses a variety of aquaporins (AQPs), with *AQP1* in microvascular endothelium and *AQP4* in large- and small-airway epithelium [55]. The AQPs function primarily as water-transporting pores to regulate water permeability. Reduced *AQP1* expression can impair water absorption in cavities, interstitium, and capillary compartments, leading to disturbed fluid transport and hypobaric hypoxia edema [56]. Moreover, *AQP1* plays an important role in angiogenesis [57]. Therefore, increasing expression of *AQP1* and *AQP4* may contribute to protecting against pulmonary edema and vascular remodeling at higher elevation.

Adhesion molecules and cadherin were predominantly up-regulated at higher elevation, with the adhesion molecule pathway also showing a significant up-regulation. Cadherins protect endothelial cells from oxidative stress-induced apoptosis [58], maintain intercellular adhesion, and regulate cytoskeletal dynamics. Changes in cell adhesion are central to angiogenesis, influencing vascular cell viability and functional state [59]. In addition, mounting studies suggest that ADAM and ADAMTS proteases facilitate tissue remodeling through matrix protein degradation and cell proliferation [60, 61]. Therefore, the up-regulation of adhesion molecule pathway suggests potential modulation of barrier integrity and vascular remodeling at higher elevation.

It should be noted that the observed metabolomic and transcriptomic differences may arise from both constitutive genetic divergence and environmentally induced plasticity in response to local thermal, hypoxic, and nutritional conditions. Seasonal acclimatization or transient physiological states could also contribute to some of the detected expression patterns. Thus, our findings should be interpreted as reflecting both adaptation and plasticity, and future studies employing controlled acclimation (normoxic/hypoxic and warm/cold) and common-garden experiments are needed to disentangle these components. Moreover, the functions and contribution of key DEGs remain to be further investigated through enzyme activity assays, protein expression, and/or histological analysis.

## Conclusion

This study revealed elevation-associated changes in plasma metabolite abundance and lung gene expression in *N. parkeri*, providing integrative molecular evidence of physiological adjustments to high-elevation environments. The widespread down-regulation of pathways related to glycolysis, the TCA cycle, oxidative phosphorylation, and fatty-acid oxidation, together with reduced plasma metabolite levels, indicates a generalized metabolic rate depression. The up-regulation of stress-related genes (*ATF3*, *JUN*, *ARNT2*), heat-shock proteins, DNA-repair factors, Wnt signaling, and ubiquitination components may contribute to cellular maintenance and stress resilience at higher elevation. Moreover, the elevation-related expression of ion channel, aquaporin, and adhesion molecule genes suggests transcriptional plasticity associated with osmotic regulation and tissue integrity. Taken together, these findings provide new insights into the physiological and biochemical adaptations of amphibians to cope with unfavorable conditions at higher elevation.

## Supplementary Information

Supplementary Material 1.

Supplementary Material 2.

Supplementary Material 3.

Supplementary Material 4.

## Data Availability

Raw transcriptomic data and metabolomic data were deposited in the Genome Sequence Archive (GSA) database (https://ngdc.cncb.ac.cn/gsa/) and OMIX (https://ngdc.cncb.ac.cn/omix) with project number PRJCA042176.
