# Supplementary material for: Elevation-associated shifts in plasma metabolite abundance and lung gene expression in the Xizang plateau frog, Nanorana parkeri
Source: BMC Genomics. 2026 Jan 20;27:193. doi: 10.1186/s12864-026-12553-w (PMC12903291; doi:10.1186/s12864-026-12553-w)

**Supplementary Figure Legends**

**Supplementary** **Fig. S1.** A metabolic network of central metabolic pathway (CMP) with 10 pathways (r_1_-r_10_) including glycolysis, pentose phosphate pathway (PPP) and TCA cycle, in which metabolites include ribulose 5-phosphate (Ru5P), glucose-6-phosphate (G6P), fructose-6-phosphate (F6P), glyceraldehyde-3-phosphate (GAP), phosphoenolpyruvate (PEP), pyruvate (PYR), and acetyl-coenzyme A (AcCoA).

**Supplementary Fig. S1**


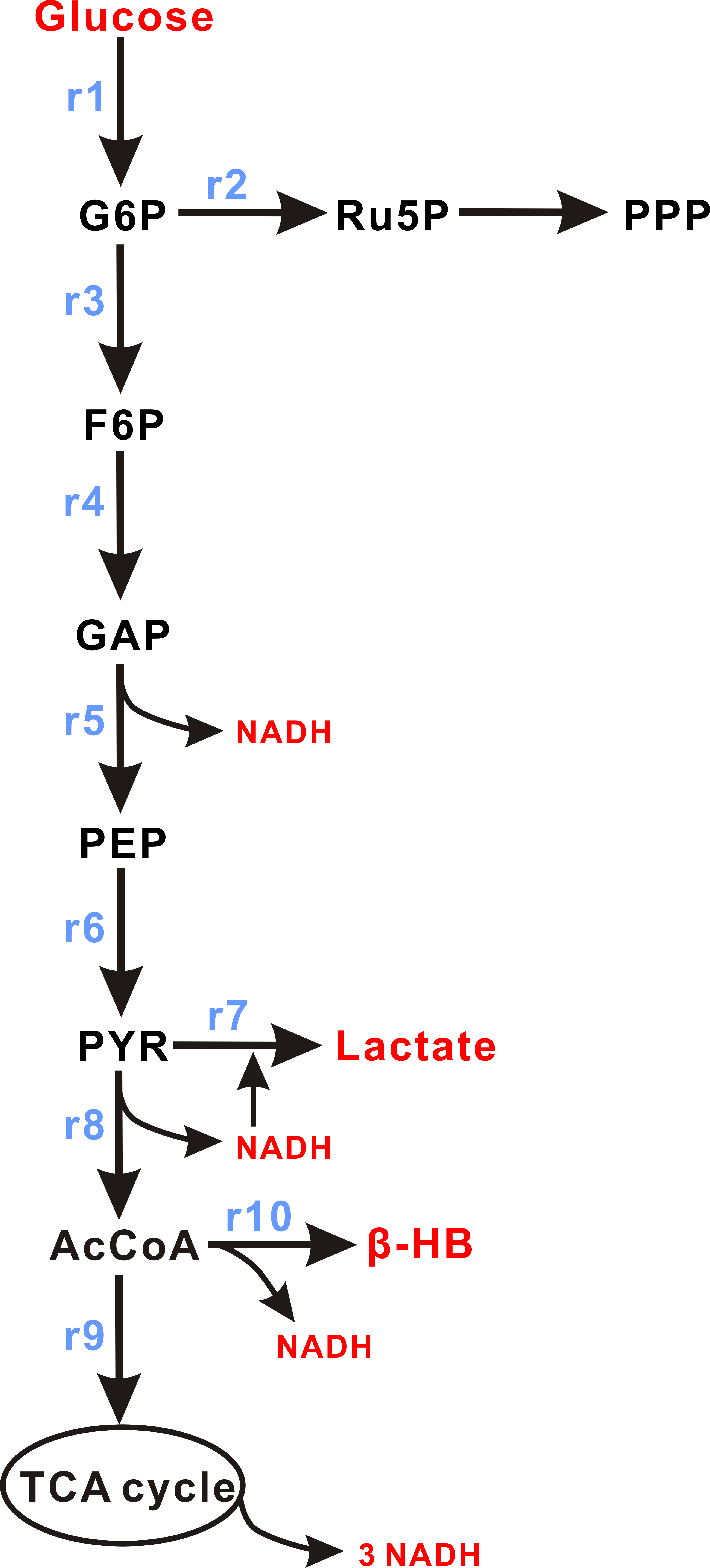

Supplement: Supplementary file 1 — Supplementary Material 1. [file 12864_2026_12553_MOESM1_ESM.doc]
